# Draft Genome Sequences of Two Virulent Serotypes of Avian *Pasteurella multocida*

**DOI:** 10.1128/genomeA.00058-12

**Published:** 2013-01-31

**Authors:** Juan E. Abrahante, Timothy J. Johnson, Samuel S. Hunter, Samuel K. Maheswaran, Melissa J. Hauglund, Darrell O. Bayles, Fred M. Tatum, Robert E. Briggs

**Affiliations:** a Department of Veterinary and Biomedical Sciences, University of Minnesota, St. Paul, Minnesota, USA; b Institute for Bioinformatics and Evolutionary Studies, University of Idaho, Moscow, Idaho, USA; c National Animal Disease Center, Agricultural Research Service, U.S. Department of Agriculture, Ames, Iowa, USA

## Abstract

Here we report the draft genome sequences of two virulent avian strains of *Pasteurella multocida*. Comparative analyses of these genomes were done with the published genome sequence of avirulent *P. multocida* strain Pm70.

## GENOME ANNOUNCEMENT

*Pasteurella multocida* is the etiologic agent of fowl cholera, a highly contagious and severe disease of poultry causing significant mortality and morbidity throughout the world (1). All types of poultry are susceptible to fowl cholera. In peracute/acute disease, death is caused by septicemia and endotoxic shock (2, 3). Serotypes A:1, A:3, and A:3,4 are most commonly associated with fowl cholera in the United States (4). The whole genome of the Pm70 strain of *P. multocida* was sequenced and annotated in 2001 (5). The Pm70 strain was isolated in 1970 from the oviduct of a chicken in Texas. This strain belongs to serotype F:3 and not A:3 as reported earlier (6) and is not virulent in chickens (7). Therefore, we sought to sequence the genomes of two virulent strains, namely *P. multocida* strains X73 (serotype A:1) and P1059 (serotype A:3). Both strains are highly virulent in turkeys, chickens, and other poultry species (8).

The genome sequencing of *P. multocida* strains P1059 and X73 was achieved via 454 pyrosequencing of a shotgun library. The sequences were assembled *de novo* with Newbler V2.6 (454 Life Sciences), and Pm70 was used as a reference strain for scaffolding; the resulting assembly generated 2.30 Mbp (40.21% GC, 24 contigs, 18 contigs >500 bp, *N*_50_ 256,544) and 2.26 Mbp (40.30% GC, 39 contigs, 28 contigs >500 bp, *N*_50_ 137,372) genomes for P1059 and X73, respectively. Genome annotation and whole genome comparisons were performed against the Pm70 strain using the RAST server (9). P1059 contained 2,144 predicted coding regions, a gene density of 88.8%, an average coding size of 961 bp, 50 tRNAs, and 4 rRNA operons. X73 contained 2,085 predicted coding regions, a gene density of 88.3%, an average coding size of 964 bp, 51 tRNAs, and 4 rRNA operons. Comparing all three strains, there were 158, 126, and 81 unique genes for strains P1059, X73, and Pm70, respectively. Multigenome comparative analysis identified the presence of a filamentous hemagglutinin *pfhB1* gene, which was highly conserved in all three strains. The *phfB2* gene was identical in X73 and P1059 strains but shared only 90% sequence identity with the Pm70 strain. A novel gene, here named *pfhB3*, was present in both X73 and P1059 but absent from Pm70. A fourth novel gene, herein named *pfhB4*, was unique to the P1059 strain. The *plpE* gene coding for a cross-protection factor antigen was present in all three strains and highly conserved (10). We identified the presence of a chondroitin synthase gene (*fcbD*) in Pm70 and the hyaluronan synthase gene (*hyaD*) in both P1059 and X73 (11, 12). Whereas the *pcgDABC* gene cluster involved in the decoration of phosphocholine to the outer core of the lipopolysaccharide (LPS) was present only in X73, the *pm1138* gene encoding a glycosyltransferase was present in all Pm70, X73, and P1059 strains (13–15).

### Nucleotide sequence accession numbers. 

The draft genome sequences of *Pasteurella multocida* subsp. *gallicida* P1059 and X73 have been deposited at DDBJ/EMBL/GenBank under the accession numbers AMBQ01000000 and AMBP01000000.
